# Teachers’ Perceptions and Preparedness for Teaching English as a Foreign Language to Students with Developmental Dyslexia: A Systematic Review

**DOI:** 10.3390/ejihpe15040064

**Published:** 2025-04-16

**Authors:** Vasiliki Folia, Afroditi Malisiova

**Affiliations:** Laboratory of Neuropsychology and Behavioral Neuroscience, School of Psychology, Aristotle University of Thessaloniki, University Campus, 54124 Thessaloniki, Greece; malisiova@psy.auth.gr

**Keywords:** developmental dyslexia, foreign language learning, teachers’ perspectives, teachers’ preparedness

## Abstract

Students with developmental dyslexia (DD) face significant challenges when learning English as a foreign language (EFL), highlighting the need for targeted support in educational systems. EFL teachers’ perceptions and preparedness regarding DD are crucial for effective instruction and improved learning outcomes in inclusive classrooms. However, no systematic review has yet explored EFL teachers’ perceptions and preparedness to teach students with DD. This systematic review, conducted in accordance with the PRISMA guidelines, examines existing research between 2005 and 2025 on EFL teachers’ perceptions and preparedness to teach students with DD. Studies were retrieved from databases including APA PsycNet, Crossref, ERIC, ProQuest, PubMed, and Scopus databases. Of 17,798 results, 16 studies met the inclusion criteria. The findings reveal mixed EFL teachers’ perceptions toward DD and inadequate training specific to DD. Moreover, practical teaching strategies and targeted interventions remain underrepresented in the literature. Most teachers lack formal DD-specific training, leading to insufficient classroom support. This review emphasizes the urgent need for improved in-service training and the development of effective resources. Future research should prioritize developing and evaluating practical teaching strategies and professional development programs on teacher preparedness in EFL contexts.

## 1. Introduction

Developmental dyslexia (DD) presents various challenges during the learning process. These challenges are particularly evident in tasks involving spelling, vocabulary acquisition, understanding complex grammar (Malisiova & Folia, 2024), interpreting oral reading and writing (Peterson & Pennington, 2015; Stein, 2018), and phonological processing (Kałdonek-Crnjaković, 2015; Malisiova & Folia, 2024; Suárez-Coalla et al., 2020).

Accumulating research evidence shows that students diagnosed with DD in their first language (L1) often experience difficulties of varying severity when learning foreign languages (FL) (Bonifacci et al., 2017; Dimililer & Istek, 2018; Kormos et al., 2019; see Kormos, 2017, 2020, for a review; Łockiewicz & Jaskulska, 2016, 2019; Nijakowska et al., 2020; Toffalini et al., 2019; Ylinen et al., 2019). This is especially true for English as a foreign language (EFL), which is commonly taught as a global lingua franca (Cimermanová, 2017; Kotzé, 2019; Lodej, 2016; Malisiova & Folia, 2024; Nijakowska, 2010). For students with DD, the same deficits that hinder their L1 learning—most notably difficulties in sound perception—also affect FL acquisition (Commissaire & Demont, 2022; Kałdonek-Crnjaković, 2015; Peterson & Pennington, 2015). These challenges adversely impact phonological awareness, making it harder to acquire new vocabulary, pronounce accurately, and comprehend spoken or written language in an FL context (Malisiova & Folia, 2024). English, in particular, poses unique challenges for DD learners due to its opaque orthography (Babuder & Jazbec, 2019; Livingston et al., 2018; Stevani & Tarigan, 2022). Irregular spelling and pronunciation, the need to memorize unfamiliar vocabulary, multiple representations of sounds, and complex grammatical structures further complicate progress in reading and spelling. These barriers significantly impact the FL learning for students with DD (Kotzé, 2019; Malisiova & Folia, 2024). Beyond academic struggles, students with DD often experience emotional challenges, including anxiety, frustration, and low self-esteem (Kormos & Smith, 2023; Malisiova & Folia, 2024).

Adapting teaching strategies to meet the needs of students with DD is essential for supporting their academic success, personal growth, and inclusion in mainstream classrooms (Kałdonek-Crnjaković, 2015; Malisiova & Folia, 2024; Malisiova et al., 2023). Teachers play a central role in creating inclusive environments and implementing educational methods for the diverse needs of students with DD (Avramidis & Norwich, 2002; Jordan et al., 2009; Malisiova & Folia, 2024; Nijakowska, 2019). However, addressing varied learning needs is demanding, especially for language teachers (Kormos & Nijakowska, 2017; Nijakowska, 2014; Worthy et al., 2016), who often lack adequate training in inclusive practices, particularly in the context of additional language instruction (Gibbs & Elliott, 2015). Research by Kormos and Nijakowska (2017) showed that language teachers’ self-confidence and attitudes toward inclusive education improved significantly after participating in a Massive Open Online Course (MOOC). Yet, teachers’ voices are often heard only when their knowledge is explicitly examined (Nijakowska, 2014). Gibbs and Elliott (2015) also found that how students are labeled (e.g., as having reading difficulties or DD) affects teachers’ sense of efficacy.

Despite growing interest, studies exploring teachers’ perspectives on DD remain limited (Han, 2015). Few focus on the challenges faced by students with DD, teachers’ perspectives, or the effectiveness of instructional interventions (Vizhi & Rathnasabapathy, 2023). Both Ferri et al. (2005) and Worthy et al. (2016) suggested that teachers draw on various sources to build their understanding of DD. However, EFL teachers often lack sufficient knowledge about how students with DD learn languages. The lack of adequate pre- and in-service training opportunities significantly impacts their perceptions about their preparedness for inclusion and can also lower motivation to provide high-quality teaching to students with DD (Nijakowska et al., 2020). Indeed, raising teachers’ awareness and offering targeted training have been shown to improve both reading skills and overall academic performance for students with DD (Vizhi & Rathnasabapathy, 2023). Adequate, research-based teacher professional training is essential for enhancing teachers’ language-based knowledge (e.g., knowledge of basic language constructs) in both L1 and FL teaching contexts (Kahn-Horwitz, 2015, 2016; Nijakowska et al., 2020; Podhajski et al., 2009; Vaisman & Kahn-Horwitz, 2020). Additionally, promoting positive teacher attitudes toward inclusive education can improve teachers’ perceptions of preparedness for inclusion (Das et al., 2013; Hsien et al., 2009; Nijakowska et al., 2020). In contrast, inadequate preparedness may foster negative beliefs about inclusion (Das et al., 2013; Nijakowska et al., 2020).

Teachers’ self-perceived preparedness to provide inclusive instruction directly influences their ability to create inclusive classrooms (Nijakowska et al., 2020). Research indicates that teachers who are more self-efficacious, experience less anxiety, and hold positive attitudes toward inclusion and students with specific learning difficulties (SLD) are more successful in implementing inclusive practices (Malisiova & Folia, 2024; Nijakowska et al., 2020; Sharma & Sokal, 2015). Teachers’ awareness of inclusive strategies, combined with knowledge of effective intervention programs and their theoretical foundations, strongly influences the level of their preparedness to teach inclusively (Das et al., 2013; Hsien et al., 2009; Kahn-Horwitz, 2015, 2016; Nijakowska et al., 2020; Podhajski et al., 2009).

To the best of our knowledge, no prior systematic reviews have specifically focused on primary and secondary EFL teachers’ perspectives on teaching additional languages to students with DD. While some literature reviews have explored DD in FL learning, none have synthesized recent research investigating EFL teachers’ perceptions and needs across diverse educational settings. In particular, prior reviews have not examined in depth how primary and secondary EFL teachers’ approaches support students with DD (Gasperini, 2022; Han, 2015; Jiang et al., 2022; Luque Sánchez, 2023). This gap in the literature is notable, given that numerous studies highlight the challenges students with DD experience in acquiring FL and the difficulties multilingual individuals with reading-related disabilities encounter at different educational levels (see Kormos, 2017 for a review). Maunsell (2020) and Černickaja and Sokolová (2024) have conducted literature reviews, not systematic reviews on DD; however, their research questions, as well as their inclusion and exclusion criteria, differ from those of the present study. Additionally, Maunsell (2020) employs a narrative review approach, while Černickaja and Sokolová (2024) adopt a scoping review methodology. Maunsell’s (2020) narrative review offers a cross-cultural and cross-linguistic perspective on dyslexia, emphasizing common international concerns and the necessity of effective teacher training. The review also highlights the importance of inclusive practices and intervention programs to enhance EFL acquisition, particularly for students with SEN, an issue that has been raised in the present review as well (Maunsell, 2020). Conversely, Černickaja and Sokolová (2024) focus on university instructors’ beliefs and perceptions regarding their attitudes toward students with DD and their awareness of dyslexia without considering primary and secondary education EFL teachers, as the present study does (Černickaja & Sokolová, 2024). However, even among university instructors, the need for practical training has been recognized (Černickaja & Sokolová, 2024). Similarly to primary and secondary educators, university teachers acknowledge the benefits of professional development programs aimed at equipping them with strategies to address the needs of students with DD effectively (Černickaja & Sokolová, 2024).

The present systematic review extends previous research by using explicit, structured methods to minimize bias and ensure reliable findings, following guidelines outlined in the Cochrane Handbook (Munn et al., 2018). It identifies and synthesizes international evidence from the past two decades on EFL teachers’ perceptions—including awareness, attitudes, and perspectives—and their preparedness, particularly their training needs, related to teaching students with DD. This review focuses on studies using quantitative methods (e.g., structured questionnaires). By following a predefined process, the present review aims to summarize global evidence on current EFL teachers’ perceptions, identify differences across educational contexts, and highlight potential gaps in the literature and/or in the current teaching practices. It also aims to inform future research directions and briefly discuss teacher education policy. Specifically, this review addresses the following key research questions: (1) What are EFL teachers’ perceptions in regard to teaching students with DD? (2) To what extent are EFL teachers prepared to teach these students, and what are their perceived training needs? What are the gaps regarding practical strategies and interventions used by EFL teachers? (3) How do EFL teachers’ perceptions and preparedness levels vary across different countries and educational contexts? By critically appraising and synthesizing findings from multiple studies, this systematic literature review aims to provide meaningful insights for teacher educators. Understanding both what is known and what remains uncertain allows for better-informed decision-making and empowers teachers to engage with greater confidence in educational discussions that often take place without their input (Worthy et al., 2016).

## 2. Materials and Methods

This systematic review followed the PRISMA statement and the process recommended by Peters et al. (2020). The PRISMA diagram was used to enhance the transparency and scientific quality of our review.

Only standardized structured questionnaires were included as an inclusion criterion to provide reliable, valid, and cross-culturally relevant insights into EFL teachers’ perceptions and preparedness for teaching students with DD. Since the reviewed papers would be internationally sourced, the concepts of perception and preparedness must be defined in a valid and reliable way. The use of standardized structured questionnaires supports validity since it accurately assesses and measures participants’ perceptions and preparedness (Taherdoost, 2016). Moreover, the choice of using such tools offers data reliability since researcher bias is minimized and consistency is maintained across contexts (Taherdoost, 2016).

In this way, it enables meaningful comparisons across educational settings. In cross-cultural research, questionnaires allow for data collection across diverse linguistic and educational contexts. For example, Nijakowska et al. (2018) and Nijakowska (2022) include participants from Greece, Slovenia, Cyprus, and Poland. Their standardized design facilitates the identification of trends and differences across cultures and nations (Nijakowska, 2022; Nijakowska et al., 2018). Moreover, questionnaires can be adapted and validated for different languages, which increases the generalizability of results. For all the above reasons, this review includes studies using only standardized, structured questionnaires.

### 2.1. Search Strategy

The papers included in this review were sourced via a systematic and manual search of relevant articles. Only papers published in the English language between 2005 and 2025 in academic journals were retrieved. The search and review of the studies were conducted from August 2024 to January 2025. A comprehensive literature search was performed using six electronic databases, namely, APA PsycNet, Crossref, ERIC, PubMed, ProQuest, and Scopus. The following search terms and their combinations were used: (“dyslexia” OR “developmental dyslexia” OR “reading difficult*” OR “learning difficult*” OR “disorder*) AND (“English”) AND (“foreign language”) AND (“teacher*” OR “educator*”) AND (“quantitative research” OR “questionnaire*”). The search string was deliberately kept broad so that no relevant articles were missed, with the expectation that irrelevant articles would be excluded later in the process.

**Figure 1:**
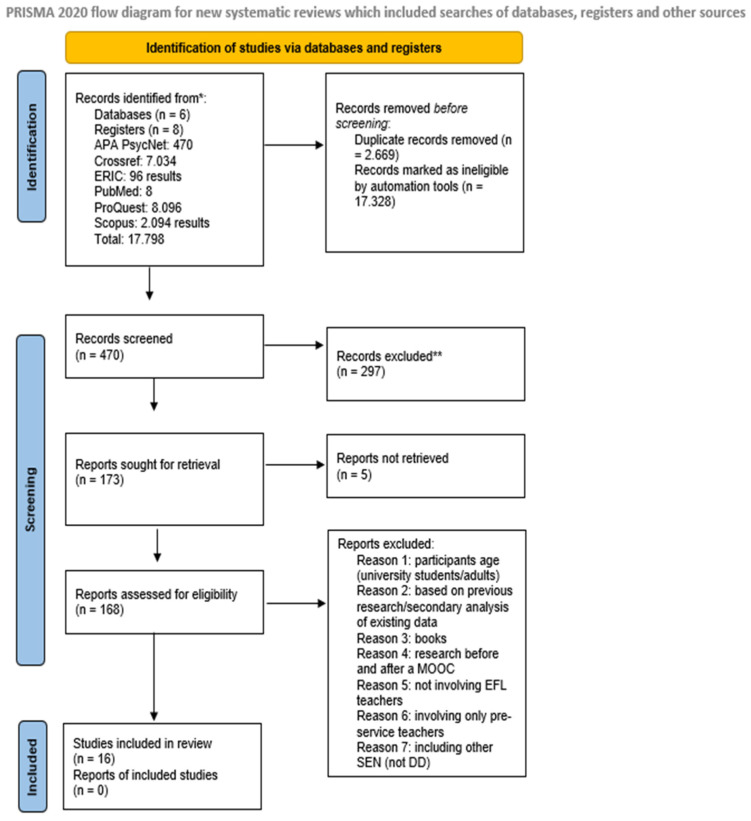
PRISMA diagram for the search protocol and the inclusion and exclusion of the reviewed articles. * We report the number of records identified from each database or register searched, rather than the total number across all databases/registers; ** No automation tools were used for the exclusion process; records were excluded manually by a human.

The initial search yielded a total of 17.798 studies published up to the 30th of January 2025. Of these, 17.328 were removed before screening, taking into consideration the relevance of the title. A total of 470 were screened based on their abstracts and keywords, and 302 were excluded for being not relevant or failing to meet the inclusion criteria. A total of 173 records were identified for retrieval, but five could not be accessed in full text. Among the 168 records reviewed, the ones that were found to focus on adults/university students, be based on previous research not presenting new findings, be books, involve research based on MOOC programs, not include EFL teachers or only pre-service teachers as participants, or refer to SEN (Special Education Needs) other than DD were excluded, resulting in a final total of 16 studies included in this review. Following the scoping review methodology, the second author supervised the process, reviewing each stage and its outcomes (Černickaja & Sokolová, 2024; Gutierrez-Bucheli et al., 2022). In case of disagreement, full records or complete texts were re-examined with particular attention to the inclusion and exclusion criteria outlined earlier (Černickaja & Sokolová, 2024). Any discrepancies in labeling between the reviewers were discussed on a case-by-case basis and adjusted accordingly. The PRISMA flow diagram summarizes this information in detail (Figure 1).

### 2.2. Eligibility Criteria

The search strategy was developed based on specific inclusion and exclusion criteria. The inclusion criteria were as follows: (1) articles published in English in peer-reviewed journals between 2005 and 2025, including proceedings of international conferences that are indexed in the databases utilized for the present review, (2) empirical research employing quantitative methods (structured questionnaires), and (3) a focus on EFL teachers’ awareness, attitudes, perspectives, and training needs regarding DD and EFL acquisition in primary and secondary education. Studies were excluded if they met any of the following criteria: (1) systematic review articles and meta-analyses, (2) empirical research employing qualitative methods (interviews, focus groups, etc.), (3) articles not addressing relevant topics such as EFL teachers’ perspectives and preparedness, (4) articles involving only pre-service teachers, (5) articles including SLD other than DD, (6) books, or (7) studies focusing on tertiary/university students.

Two researchers independently reviewed the citations during the title, abstract screening, and full-text review phases. Possible discrepancies were resolved through discussion and consensus.

## 3. Results

### 3.1. Study Characteristics

The final review resulting from the screening processes included a total of 16 articles related to EFL teachers’ perceptions and attitudes toward students with DD, their awareness and training regarding DD, the challenges of including DD learners, and the implementation and integration of appropriate teaching practices and approaches. Table 1 presents the study characteristics of the reviewed articles in chronological order. When multiple articles were published in the same year, they are listed alphabetically by the authors’ surnames. Most of the research was conducted within the past five years (76.47%). A total of 2.519 FL teachers participated in the studies by completing questionnaires that explored their perceptions and preparedness. Regarding the study settings, research was conducted in various countries, notably, three studies each in Greece and Poland, while two studies were conducted in Algeria and Turkey.

### 3.2. Tools Used to Explore Teachers’ Awareness, Attitudes and Perspectives, and Training Needs

The questionnaires used by the literature were either standardized or developed by the researchers for the specific studies and were mainly distributed online. Nijakowska et al. (2018) developed a new questionnaire based on the DysTEFL-Needs Analysis Questionnaire (Gibbs & Elliott, 2015). This revised tool, the DysTEFL-Needs Analysis Questionnaire Revised (DysTEFL-NAQ-R), measures pre- and in-service EFL teachers’ beliefs about their preparedness to include DD learners in mainstream EFL classrooms called TEPID (Teacher of English Preparedness to Include Dyslexics) and assesses their professional training needs regarding DD and inclusive instructional practices. Özçelik and Elverici (2024) also employed the DysTEFL-NAQ-R, adding a fourth section for teachers’ additional comments.

Kumas et al. (2021) employed “The Development of the Teachers’ Beliefs About Literacy Questionnaire” (TBALQ), originally created by Westwood et al. (2005), to identify teachers’ teaching approaches and their beliefs about early literacy acquisition and methods for teaching early readers. Similarly, Nushi and Eshraghi (2023) employed “The Knowledge and Beliefs about Developmental Dyslexia Scale” (KBDDS), by Soriano-Ferrera and Echegaray-Bengoa (2014), which uses a 36-item scale to evaluate EFL teachers’ awareness of DD. Nijakowska (2022) used a questionnaire adapted from the FLIPD—Perceptions about Inclusive Practices in Teaching Foreign Languages to Dyslexic Language Learners—in pre- and post-course surveys. This 24-item instrument was designed to assess the participants’ attitudes towards inclusion in FL education, their self-efficacy beliefs, and their concerns about implementing inclusive FL instructional practices for DD students.

The rest of the reported studies designed self-constructed questionnaires based on pertinent literature on the area of DD, EFL acquisition, and teacher education (Babuder & Jazbec, 2019; Cimermanová, 2017; Fekih, 2019; Haggag & Bakr, 2020; Indrarathne, 2019; Kalsoom et al., 2020; Lemperou et al., 2011; Mohammad, 2022; Oskwarek et al., 2024; Tobbi, 2020; Vizhi & Rathnasabapathy, 2023). They investigated various topics such as teachers’ understanding of fundamental concepts, intervention strategies, awareness, classroom challenges, and assessment methods related to DD. The reliability and validity of these tools were established through evaluations by external experts and/or a piloting phase (Babuder & Jazbec, 2019; Nijakowska et al., 2018; Tobbi, 2020).

### 3.3. EFL Teachers’ Awareness Regarding Developmental Dyslexia

Although research indicates that challenges associated with DD extend into EFL learning, teachers often remain underinformed and undertrained at this intersection. Özçelik and Elverici (2024) found that while most FL teachers are aware of DD and try to support students with DD, they frequently feel unprepared due to limited training and resources. Out of a sample of 180 participants, 8.9% of teachers did not believe in maintaining high expectations for DD learners, reflecting a lack of awareness about their potential. Additionally, they found that 59.4% of teachers stressed the importance of family collaboration, and over 70% supported mainstream inclusion. However, only 16.7% reported understanding the specific challenges DD learners face, and 8.9% were unsure how to assist them. This highlights a lack of confidence in delivering differentiated instruction (Özçelik & Elverici, 2024). Notably, 75% of teachers reported receiving no education on EFL and DD during their formal training. Despite this, many expressed a strong interest in learning more about DD, effective teaching strategies, and supportive assessment techniques (Özçelik & Elverici, 2024).

Nushi and Eshraghi (2023) assessed DD awareness among 84 Iranian English teachers at language institutes. Their findings confirmed low awareness levels, which hinder effective support for students with DD. Nonetheless, teachers expressed interest in expanding their knowledge of DD. Similar results were reported by Tobbi (2020), who found that Algerian middle school EFL teachers generally learned about DD through personal efforts rather than formal education. In the same vein, Fekih (2019) noted widespread confusion between DD and other disabilities among Algerian EFL teachers. Notably, 40 out of 42 middle school teachers reported never encountering the term “reading impairment”. Fekih (2019) further highlighted that, despite evidence that teacher awareness plays a crucial role in student success, most EFL teachers remain unfamiliar with DD. This lack of awareness extends to parents as well. Fekih (2019) called for national conferences and workshops to raise awareness and provide essential guidance.

In contrast, Lemperou et al. (2011) found that most Greek EFL teachers, from a sample of 94 participants, were aware of the challenges faced by students with DD when learning EFL, primarily due to their experience teaching them in mainstream classrooms. Nonetheless, these teachers also felt poorly equipped to meet these challenges due to limited knowledge of suitable teaching strategies.

Overall, the studies in this systematic review reveal a clear pattern: EFL teachers often lack sufficient awareness and preparation to support students with DD. While some show general awareness of DD and are willing to support DD students, most feel undertrained due to their limited formal training and resources.

### 3.4. Cross-Country Comparisons of EFL Teachers’ Awareness Levels of Developmental Dyslexia

Research on teachers’ awareness of DD across different countries reveals significant variations (Table 2). The main finding is that many educators across countries lack adequate knowledge of SLD. In Sri Lanka, Indrarathne (2019) found that 94% of teachers did not have a clear understanding of DD, and this indicates a severe gap in awareness. Similarly, in Algeria, Fekih (2019) and Tobbi (2020) reported that teachers had poor knowledge of DD, with the latter study revealing that only 11.11% of participants were aware of this reading difficulty. A similar pattern was observed in Iran, where Nushi and Eshraghi (2023) found that most EFL teachers lacked sufficient understanding of DD. Their awareness was independent of demographic factors such as gender, education, and experience. Likewise, in Pakistan, Kalsoom et al. (2020) concluded that most teachers had little awareness of students’ learning difficulties, mirroring findings from Algeria and Sri Lanka.

In contrast, studies from European countries present a slightly better picture. In Greece, Lemperou et al. (2011) found that 51% of EFL teachers were aware of DD and its challenges, while 45% had limited knowledge. This suggests a more balanced distribution of awareness levels compared to the near-total lack of understanding observed in Sri Lanka and Algeria (Indrarathne, 2019; Tobbi, 2020). A later study by Nijakowska (2022) in Greece, Slovenia, and Poland revealed that 63.6% of teachers rated their knowledge as average, 31.9% as poor, and 11.6% as good. These findings indicate that while awareness is present in some European countries, significant gaps remain. Further supporting this, Oskwarek et al. (2024) found that most FL teachers in Poland were aware of DD, highlighting a relatively higher level of knowledge compared to non-European regions.

Overall, while awareness of DD is higher in some European countries, significant gaps remain—particularly in non-European regions—such as Sri Lanka, Algeria, and Pakistan, where knowledge of the condition is severely lacking. Even in contexts where awareness is relatively better, studies consistently highlight the need for further professional development to ensure teachers are adequately prepared to support learners with DD. However, since the research presented above involves only thirteen countries worldwide that have studied this topic so far, it is necessary to conduct research in more countries to achieve more valid and reliable cross-country comparisons.

### 3.5. EFL Teachers’ Attitudes and Perspectives on Students with Developmental Dyslexia

The study by Babuder and Jazbec (2019) suggests that most in-service FL teachers recognize DD as an essential topic. However, some teachers exhibit negative perceptions of the term “developmental dyslexia” and hold lower expectations for students with DD. This can lead to unequal treatment compared to their peers. Such disparity may stem from misunderstandings and a limited awareness of DD (Fekih, 2019; Indrarathne, 2019; Özçelik & Elverici, 2024; Tobbi, 2020) Some teachers believe that high expectations are unnecessary for DD learners, reflecting a lack of understanding of their strengths and potential (Fekih, 2019). These misconceptions can result in assumptions that DD learners are less capable or lack motivation. In some cases, they have even been labeled as “lazy” or “unmotivated” and referred for administrative intervention, increasing the risk of academic failure (Fekih, 2019).

Moreover, teachers often hold certain misconceptions about DD, frequently associating it with visual processing issues, such as perceiving letters in reverse, rather than understanding its neurological and cognitive underpinnings, according to Kumas et al. (2021). Although they often rely on conventional reading instruction, they acknowledge the importance of tailored support and are eager for additional training (Kumas et al., 2021). This willingness for further training on DD in general reflects a positive attitude toward implementing effective, evidence-based teaching strategies (Kumas et al., 2021). However, because DD is not immediately visible, unlike some other disabilities, teachers may not prioritize learning about it (Fekih, 2019). Furthermore, a significant portion of teachers reports negative experiences when teaching students with DD (Fekih, 2019). They also identified the inadequacy of the EFL Cross-Thematic Curriculum Framework and current textbooks as significant barriers to supporting these students effectively in mainstream classrooms (Lemperou et al., 2011). Research highlights that training programs could improve teachers’ attitudes toward DD, enhancing their understanding and preparedness to implement inclusive practices (Indrarathne, 2019). Studies reveal that trained teachers generally hold supportive attitudes toward DD learners and recognize the importance of adopting a differentiated and multisensory approach (Oskwarek et al., 2024).

In conclusion, many EFL teachers acknowledge the importance of addressing DD. However, prevailing misconceptions and limited understanding often result in lower expectations and inadequate support for these students. Particularly of note, when teachers attribute learning difficulties to laziness or lack of motivation and use ineffective teaching methods, they further hinder the academic progress of learners with DD.

### 3.6. EFL Teachers’ Training Needs

Additionally, the focus of recent research has extended to EFL teachers’ training and professional development needs (Lemperou et al., 2011; Nijakowska et al., 2018; Nushi & Eshraghi, 2023; Tobbi, 2020). Despite the pressing need for professional training, many EFL teachers still lack sufficient understanding and access to training opportunities (Mohammad, 2022). Studies consistently report a significant underdiagnosis of DD, largely due to the absence of nationwide screening programs, as evidenced by Ghonsooly and Javadian (2010).

The study conducted by Babuder and Jazbec (2019), involving 34 English language teachers in Iraqi primary schools, found that while these teachers generally possessed theoretical knowledge of DD and were open to further training, few had practical experience teaching a foreign language to DD students. Using a three-point Likert scale questionnaire and open-ended questions, the study underscored the urgent need for specialized training to help EFL teachers identify and support students with DD in EFL contexts (Babuder & Jazbec, 2019). Similarly, Kalsoom et al. (2020) noted that while teachers are familiar with the term “developmental dyslexia” and its challenges, there is a clear need for enhanced support and structured guidance.

Additionally, Indrarathne (2019) identifies a significant gap in EFL teachers’ understanding of learning difficulties and inclusive language teaching. In a study involving 129 participants, Indrarathne (2019) emphasized the need for English language teaching (ELT) training that includes both theoretical content and practical applications. Institutional, social, and cultural factors were found to significantly influence the classroom implementation of inclusive practices. Moreover, the study reported a lack of formal training on DD and reading disorders, with only a small percentage of teachers having received training in these areas (Indrarathne, 2019). Cimermanová (2017) identified critical gaps in special education training among in-service teachers. Specifically, out of 243 EFL teachers (187 in-service and 56 pre-service), 34.9% had received no special education training (Cimermanová, 2017). Additionally, 58.6% had not included students with SEN in their classrooms (Cimermanová, 2017). Nevertheless, 41.4% of teachers reported currently working with SEN students, and over 45% suspected that undiagnosed SEN students were present in their classrooms (Cimermanová, 2017).

Nijakowska et al. (2018) also investigated the impact of intensive training on in-service EFL teachers’ self-efficacy, concerns, and attitudes toward inclusive practices. Using pre- and post-training self-report surveys, they found that even short, intensive courses—whether online or in-person, and ideally certified by universities—could significantly enhance teachers’ skills and knowledge in supporting DD learners (Nijakowska et al., 2018). The authors advocate for in-service training (INSET) courses that provide realistic insights into DD and offer practical teaching. Such training would enable EFL teachers to help DD students overcome learning challenges, develop awareness of reading and writing processes in the target language, build self-esteem, increase motivation, and fully integrate into the EFL classroom (Nijakowska et al., 2018). They also emphasize the need for adequate resources, examination support, appropriate teaching approaches, assessment tools, curriculum differentiation, classroom management, and parental involvement (Nijakowska et al., 2018).

Fekih (2019) further emphasizes the urgent need for enhanced teacher training through national conferences and workshops. Nijakowska (2022) found that teachers with prior training started with higher self-efficacy, though differences diminished post-course. Initial experience with DD students reduced concerns before the course, but this effect lessened post-course. Interestingly, teacher trainers and higher education professionals showed increased concerns despite gains in self-efficacy and knowledge (Nijakowska, 2022). The course in Nijakowska (2022) significantly improved the participants’ perceived knowledge of DD. Demographic analysis revealed that pre-course knowledge of DD positively influenced initial self-efficacy and concerns, while post-course perceived knowledge strongly correlated with post-course self-efficacy. General teaching experience, employment status, and education level had no significant effect. However, age influenced pre-course but not post-course attitudes. Concerns varied according to teaching context and employment status, with Polish teachers and those in higher education expressing greater concerns. The study underscores the potential benefits of intensive training to enhance teachers’ preparedness for inclusive practices and calls for further research on how these training effects translate into classroom practices and student outcomes (Nijakowska, 2022).

Haggag and Bakr (2020) further emphasized the need for enhanced training in intervention techniques and assessment practices. They highlighted the importance of teacher preparedness in addressing learning difficulties. Other studies focusing mainly on teacher perceptions (Lemperou et al., 2011; Özçelik & Elverici, 2024) also found that many teachers viewed recommended teaching methods as ineffective for DD students. These studies underscored the importance of collaboration between teachers and families, echoing research that supports family–school partnerships in addressing DD.

In conclusion, the reviewed studies underscore the critical need for systematic and well-structured INSET courses. Such courses are expected to improve EFL teachers’ knowledge and ability to support learners with DD. While many teachers demonstrate theoretical awareness and a willingness to learn, the lack of practical experience, formal training, and institutional support hinders inclusive teaching practices. Well-structured INSET programs, including practical strategies, awareness-building, and collaboration with families, are essential to bridging this gap.

### 3.7. EFL Teachers’ Training on Developmental Dyslexia Across Different Countries

Research on teacher training regarding DD highlights significant gaps across various countries (Table 3). The main finding is that most educators lack sufficient preparation to support DD learners. In Sri Lanka, Indrarathne (2019) found that none of the teachers had received training on accommodating DD students, indicating a critical need for professional development. Similarly, in Algeria, Fekih (2019) reported that teachers’ negative attitudes toward DD were linked to inadequate training and awareness. Tobbi (2020) found that 93.83% of Algerian teachers believed they needed and would benefit from training programs. Likewise, in Turkey, Kumas et al. (2021) found that 95% of teachers had not received training on SLD, and those who had been trained found the training insufficient, reflecting similar challenges in teacher preparedness.

European studies present a slightly better picture, yet they still highlight the necessity for additional training. In Greece, Lemperou et al. (2011) found that while 87% of teachers expressed strong interest in INSET, only 2.1% had received in-school training. In Slovakia, Cimermanová (2017) reported that 34.9% of teachers had no special training in teaching students with special educational needs (SEN). However, some had pursued alternative professional development opportunities, with 9% taking courses of 60+ hours and 6% completing 30–60 h of training. In Slovenia, Babuder and Jazbec (2019) reported that 63.6% of in-service teachers and 40.4% of pre-service teachers preferred a short, two-day training program. On the other hand, a smaller proportion (8.1% of in-service and 9.6% of pre-service teachers) supported one-year specialist training. Meanwhile, Nijakowska et al. (2018) found that in Greece, Cyprus, and Poland, 90.7% of the participants showed some interest in training, and 53.3% were resolute about joining professional development. In Poland, Nijakowska (2022) found that only 39.1% of teachers had received previous training on teaching DD learners, implying that 60.9% remained untrained.

Despite the limited training available, research consistently shows teachers’ willingness to learn. In Iran, Nushi and Eshraghi (2023) found that the majority of teachers expressed a desire to learn more about DD. Similarly, in Turkey, Özçelik and Elverici (2024) reported that 50.6% of teachers wanted to increase their knowledge, 34.4% recognized their need for more information, 41.1% were interested in additional training, and 37.2% were committed to enrolling in professional development courses. It is worth mentioning that even in countries where awareness is more pronounced, a need for further education persists. In Greece, Cyprus, and Poland, Nijakowska et al. (2018) found that 94.4% of teachers felt they needed more information on effective language teaching methods for DD learners. Similarly, in Slovenia, Babuder and Jazbec (2019) reported that while teachers had theoretical knowledge of dyslexia, few had direct teaching experience with dyslexic students. A comparable pattern was observed in Turkey, where Özçelik and Elverici (2024) found that 8.9% of teachers did not believe English teachers should set high expectations for dyslexic learners, suggesting misconceptions about their potential.

Overall, the findings suggest that teachers across different countries do acknowledge their need for training. However, actual opportunities for professional development remain limited. Non-European countries, such as Sri Lanka, Algeria, and Turkey, report an almost complete lack of structured training, whereas European countries show a slightly better, yet still insufficient, level of professional development. The strong interest in training across all countries underscores the urgent need for educational policies that prioritize teacher training in DD-inclusive pedagogy. Moreover, more research is required to provide evidence-based findings that will lead to more reliable cross-country comparisons.

## 4. Discussion

### 4.1. Key Findings and Theoretical Contributions 

To our knowledge, this review is the first to systematically examine how EFL teachers perceive and prepare for teaching DD learners, with a focus on their awareness, attitudes, perspectives, and training needs. Of the 17,798 studies evaluated, 16 met the inclusion criteria. Our systematic review expands the paradigm of inclusive education into the often-overlooked domain of EFL instruction. In addressing research question (1), we found that EFL teachers’ perceptions of teaching students with DD are mixed. While many EFL teachers are aware of DD and willing to support such learners, they lack the necessary training and resources. Similarly, many EFL teachers recognize the importance of addressing DD; however, the prevailing misconceptions and limited understanding often lead to lower expectations and insufficient support for students with DD. With regard to research question (2), which pertains to teachers’ preparedness, we identified significant gaps in formal training and a lack of practical strategies specifically tailored to inclusive EFL instruction. With regard to research question (3), the findings of (1) and (2) are similar across all countries examined, with a slightly better picture presenting in European countries. The key themes or trends emerging from the reviewed studies included a lack of DD-specific formal training, a pressing need for improved in-service training and resources, and the importance of developing practical teaching strategies through targeted professional development programs.

Our review offers a theoretical contribution by bridging the fields of EFL, inclusive education, and teacher cognition. It provides a conceptual framework illustrating how EFL teachers’ beliefs, knowledge, and training intersect to influence inclusive pedagogical practices. There is variability in teacher perceptions, which underscores that they are not fixed across countries. Factors that can shape such variability are sociocultural factors, such as institutional culture and access to training (Zhou, 2024). Moreover, this review identifies a theoretical blind spot in the intersection of language, cognition, and disability in EFL contexts. Teachers’ awareness of DD often lacks depth, particularly concerning its impact on second language acquisition and cognitive processing (Forlin, 2013; Gibbs & Elliott, 2015; Sharma et al., 2015). In multilingual and multicultural settings, limited awareness can further hinder effective instruction (Coyne et al., 2013).

Teachers’ attitudes and perspectives toward DD learners are diverse and often influenced by misconceptions. Some still believe that students with special education needs, including DD, are better served in specialized settings (Forlin, 2013; Sharma et al., 2015), while others hold lower expectations based on the inaccurate belief that DD equates to low motivation or limited ability (Babuder & Jazbec, 2019; Fekih, 2019; Indrarathne, 2019; Özçelik & Elverici, 2024). Limited awareness and knowledge contribute to negative perceptions and impede effective interventions (Nushi & Eshraghi, 2023; Tobbi, 2020). These beliefs significantly affect instructional approaches, with many doubting their ability to teach DD students effectively due to skill gaps (Gibbs & Elliott, 2015). Even well-informed teachers frequently feel ill-equipped to teach DD learners (Lemperou et al., 2011).

In terms of preparedness and training, most EFL teachers demonstrate significant gaps in both theoretical understanding and practical experience with students with DD (Babuder & Jazbec, 2019). While some possess foundational knowledge, few feel confident in implementing inclusive strategies tailored to the cognitive–linguistic demands of EFL instruction settings (Forlin, 2013; Sharma et al., 2015). This highlights a critical theoretical insight: generalized special education training is inadequate for preparing teachers in subject-specific contexts like EFL. Inclusive practices must be adapted to the unique challenges of second language learning, including decoding, phonological processing, and syntactic awareness—areas where many teachers report feeling underprepared (Forlin, 2013; Gibbs & Elliott, 2015; Kumas et al., 2021; Sharma et al., 2015). Consistently across studies, there is a clear lack of sufficient pre- and in-service training on inclusive teaching practices and special education (Babuder & Jazbec, 2019; Cimermanová, 2017; Indrarathne, 2019; Sharma et al., 2015; Tobbi, 2020). Most teachers report no formal preparation in special education (Cimermanová, 2017), while others identify major gaps in understanding how to support DD learners in language classrooms (Indrarathne, 2019). Yet, evidence from the review shows that intensive and well-designed training programs can improve teachers’ self-efficacy and inclusive teaching capabilities (Haggag & Bakr, 2020; Nijakowska, 2022; Nijakowska et al., 2018). Incorporating current research into ongoing professional development is essential for equipping teachers with effective teaching strategies and for fostering inclusive classroom environments (Forlin, 2013; Kalsoom et al., 2020; Sharma et al., 2015).

All of the above is particularly important when considering the reciprocal relationship between professional development, teachers’ beliefs in their preparedness, and their instructional effectiveness, which is well documented in the literature (Holzberger et al., 2013; Tzivinikou, 2015; Velthuis et al., 2014). Research indicates that both pre-service (PRESET) and INSET programs play a crucial role in supporting teachers’ self-efficacy and instructional quality by strengthening teachers’ confidence and competency (Velthuis et al., 2014). Self-efficacy has been identified as a long-term predictor of instructional quality, particularly in fostering a supportive classroom climate and effective instructional strategies (Velthuis et al., 2014). Research further supports that continuing professional development (CPD) significantly influences teaching effectiveness, which bears a significant impact on teachers’ self-efficacy (Tzivinikou, 2015). Thus, teachers who experience growth in self-efficacy through training are more likely to apply newly acquired teaching strategies in practice, thereby improving instructional quality (Holzberger et al., 2013; Tzivinikou, 2015). The literature also suggests a reciprocal relationship between self-efficacy and instructional quality, as higher self-efficacy correlates with student achievement, motivation, and teachers’ job satisfaction and well-being (Holzberger et al., 2013). Additionally, teachers with a heightened awareness of self-efficacy typically exhibit a stronger commitment to their profession, greater perseverance, resilience when encountering challenges, and a deeper understanding of students’ diverse needs (Nijakowska et al., 2018). They are more likely to implement innovative teaching methods, set higher learning goals for students, and promote their autonomy (Holzberger et al., 2013). Consequently, all the above prerequisites could lead to more supportive educational environments for SEN and SLD students.

Consequently, our review contributes to a developing theory of professional learning in inclusive education. It shows that effective training must be both contextually relevant and cognitively aligned. In the case of EFL teachers, this means integrating insights from special education with foreign language acquisition principles. Ultimately, the present review affirms that inclusive EFL education requires subject-specific pedagogical knowledge supported by robust, evidence-based training frameworks.

### 4.2. Limitations of the Included Research Articles and This Systematic Review

The studies reviewed in the present systematic review have several limitations. Teachers’ perceptions and preparedness were measured solely through self-reports introducing potential biases that could distort assessments (Nijakowska et al., 2018), without verification via classroom observations (Basturkmen, 2012). This reliance risks over- or underestimating competencies, as self-reports may not align with actual practices. To overcome these challenges, future research can address self-report bias by integrating objective and diverse data collection methods. For example, incorporating mixed-method approaches, such as direct classroom observations, structured interviews, or written reflections, could enhance reliability. Additionally, longitudinal studies could offer deeper insights by tracking changes in teacher perceptions and preparedness over time in order to reveal long term patterns and effects of training interventions.

Furthermore, the small sample sizes in studies by Fekih (2019), Mohammad (2022), and Vizhi and Rathnasabapathy (2023) (*n* = 42, *n* = 34, *n* = 19, respectively) reduce factor analysis stability and limit the verification of validity interventions (Vizhi & Rathnasabapathy, 2023). Variations in demographics such as gender, school type, and experience were unexplored due to uneven response distribution (Mohammad, 2022; Nijakowska et al., 2018). The demographic homogeneity—primarily female EFL teachers—further constrains generalizability (Nijakowska et al., 2018; Özçelik & Elverici, 2024). For example, Özçelik and Elverici’s (2024) study focused mainly on Moroccan women, potentially biasing results. Notably, Özçelik and Elverici (2024) studied only Ministry of National Education (MoNE) language teachers, excluding tertiary-level instructors. Broader representation across genders, regions, and education levels is needed (El Arbaoui, 2023). Limited school participation further affects results. Vizhi and Rathnasabapathy (2023) studied one primary school, while Mohammad (2022) included only 12 primary schools (five public and seven private) due to COVID-19 restrictions, with data collected over a short 14-day period via email interventions. Convenience sampling and single-country focus reduce generalizability, contrasting with Nijakowska et al.’s (2018) broader cross-country study (three countries).

Our review also has some methodological limitations. It includes only peer-reviewed studies from six databases, introducing potential publication bias. Broader searches could have identified more studies. Inconsistent definitions of teacher awareness, attitudes, perspectives, training needs, and inclusive practices complicate comparisons, especially given the varied research areas, including EFL teachers’ perceptions of students with DD. Only studies from 2005 to 2025 were included to reflect recent trends, but this may have excluded older, relevant studies. Finally, restricting this review to English-language articles may have also excluded valuable research in other languages. Despite these limitations, this review offers important insights into EFL teaching in primary and secondary education and teachers’ perceptions and preparedness.

### 4.3. Practical Implications for EFL Teaching in Students with DD

This review offers both practical and theoretical contributions to the field of EFL education for learners with DD. Most notably, it challenges the traditional separation between special and mainstream education. Our findings reveal the need for a more integrated, inclusive model of language pedagogy. Developmental dyslexia belongs within the continuum of learner diversity, and this is an essential dimension that EFL teachers must be equipped to address. This conceptual shift contributes to a broader theoretical reorientation—from a deficit-based to a strengths-based approach, neurodiversity-oriented framework. Such a framework is already being explored and applied in work with other child populations, such as those on the autism spectrum, in addition to DD (Donaldson et al., 2017). The studies reviewed consistently demonstrate that DD learners exhibit a wide range of strengths and challenges, making simplistic generalizations ineffective. Teachers often misinterpret these difficulties, highlighting the need for better awareness and effective remedial strategies (Fekih, 2019; Indrarathne, 2019; Özçelik & Elverici, 2024; Tobbi, 2020). For example, structured instruction in students’ native language and monitoring in EFL classrooms have been shown to reduce learning barriers (Ashcraft, 2006). These findings reinforce the need for inclusive teaching frameworks (Rogahang et al., 2024). The findings from the reviewed studies also have several practical implications that require policy responses. Educational institutions and governments should consider revising teacher training curricula to include compulsory modules on DD and second language acquisition, with a focus on evidence-based, inclusive teaching strategies (Effendi et al., 2024; Nijakowska, 2019). Policymakers and educational institutions should prioritize training programs addressing the needs of DD students, since approximately 70–80% of students with SLD exhibit DD, representing 5–10% of the global population and 5–15% of the school population (Forteza et al., 2018; Malisiova & Folia, 2024; Maunsell, 2020). Moreover, governments and ministries of education must integrate DD-focused training into both PRESET and INSET programs. Given the reported deficiencies in existing INSET programs, such as lack of continuity and coherence (Commission of the European Communities, 2007), there is a pressing need for reforms that emphasize both theoretical knowledge and practical application (Avalos, 2011). Collaborative INSET programs could potentially ensure teacher preparedness and promote inclusive FL instruction within state schools.

#### 4.3.1. Teacher Training and Inclusion

A key theoretical insight emerging from this review is the redefinition of inclusive competence as a core component of EFL teacher expertise. Currently, training in inclusive education is often peripheral and is treated as a supplementary rather than foundational element of teacher development. This review proposes a conceptual model where inclusive education is integral to EFL training. Specifically, it calls for curriculum design that proactively implements cognitive neurodiversity (Donaldson et al., 2017) rather than fitting inclusion into pre-existing structures. Empirical evidence from the studies reviewed points to a critical need for specialized INSET focused on DD learners (Beaton, 2004; Lemperou et al., 2011). Teachers are frequently left to adapt general curricula on their own due to the lack of resources tailored to DD learners (Lemperou et al., 2011; Schneider & Crombie, 2003). Effective professional development should include explicit instruction in reading, writing, spelling, and pronunciation, equipping educators with tools to support diverse learners (Indrarathne, 2019; Rogahang et al., 2024). Here, the theoretical contribution lies in highlighting that curriculum development must be reconceptualized to address cognitive diversity proactively rather than reactively.

This review also supports transformative learning theory, which states that educators’ beliefs and their identities evolve through critical reflection and practical experience (Leaver et al., 2021; Mei et al., 2022). Exposure to hands-on strategies such as phonological awareness training, explicit reading instruction, and culturally responsive methods fosters shifts in teacher perceptions about learners with DD (Das et al., 2013; Luque Sánchez, 2023). This reflects a deeper theoretical movement toward viewing teacher development as an ongoing, reflective process shaped by experience. As shown in studies (Fekih, 2019; Kormos & Nijakowska, 2017; Nijakowska, 2022; Nijakowska et al., 2018), targeted INSET courses can enhance teachers’ efficacy and promote inclusive practices. Theoretical frameworks related to teacher learning gain empirical support here: EFL teachers are more prepared to create inclusive classrooms when training is directly linked to the challenges they encounter in their specific teaching contexts.

Finally, improved teacher preparedness not only enhances instruction but also has profound effects on students’ self-esteem and motivation in EFL settings (Lemperou et al., 2011). It has been shown that teachers’ preparedness improves with direct experience of teaching EFL students with DD (Nijakowska, 2022; Nijakowska et al., 2018). This underscores the bidirectional relationship between theory and practice to shape better educational outcomes. EFL teacher education programs must therefore integrate learning difficulties and inclusive education into workshops, courses, and CPD activities (Acar et al., 2023).

#### 4.3.2. Global Collaboration and Best Practices

From a theoretical standpoint, this review supports a global–local model for inclusive EFL education. The necessity for culturally responsive and context-specific training is particularly salient in multilingual settings (Holzberger et al., 2013).

While national policies often shape pedagogical practices (Kumas et al., 2021), international collaboration could improve resource sharing and standardize training practices globally (Coyne et al., 2013); therefore, this model argues for creating international best practices within local frameworks contributing to an emerging theory of culturally adaptive pedagogy (McArthur, 2010; Tibenda et al., 2021). In response, teachers increasingly employ learner-centered approaches, assistive technologies, and multi-sensory instruction, often collaborating with other professionals to meet students’ needs (Oskwarek et al., 2024; Vizhi & Rathnasabapathy, 2023). These practices align with constructivist theories of learning that emphasize active engagement and multi-sensory input (Taber, 2018; Volpe & Gori, 2019; Zajda, 2021).

Early identification and tailored teaching are crucial for improving outcomes (Vizhi & Rathnasabapathy, 2023). Assessment practices now emphasize developing speaking and writing skills through tools like thematic dictionaries, mind-mapping, and sentence creation activities. Gap-filling exercises enhance listening skills, while open discussions strengthen speaking abilities (Oskwarek et al., 2024). Theoretical development in this area should focus on bridging policy research with classroom-level inquiry. This will help in understanding how top–down policies interact with bottom–up teaching innovations.

#### 4.3.3. Future Research Directions

Future research should further explore EFL teachers’ perceptions and preparedness when working in mixed-ability classrooms, particularly regarding students with DD. Studies should include diverse samples across countries and employ mixed methods to ensure validity and generalizability (Gibbs & Elliott, 2015; Vizhi & Rathnasabapathy, 2023). Moreover, future research should examine how DD uniquely affects multilingual learners in EFL contexts. As noted, English has a deep orthography and this poses particular challenges for learners with DD, especially those whose L1 has a more transparent orthography (e.g., Italian or Greek) (Folia et al., 2023). Furthermore, many multilingual learners lack strong foundational literacy in their L1 (Bonifacci et al., 2017; Dimililer & Istek, 2018; Kormos et al., 2019; Kormos, 2017, 2020; Łockiewicz & Jaskulska, 2016, 2019; Nijakowska et al., 2020; Toffalini et al., 2019; Ylinen et al., 2019), which is crucial for second language acquisition. Therefore, future studies should investigate the cognitive and linguistic variables that mediate these effects.

Secondly, studies should assess the long-term impact of teacher training in inclusive education. It is essential to determine whether such interventions lead to sustained changes in teacher perceptions and student outcomes over time. As stated earlier in our results, existing evidence so far suggests training can improve teacher attitudes toward DD.

Third, research should explore the role of digital tools and platforms in supporting DD learners in EFL classrooms. As stated earlier, trained teachers often emphasize the need for differentiated, multisensory instruction (Indrarathne, 2019), yet many feel underprepared to employ such tools in areas such as decoding, phonological processing, and syntax (Forlin, 2013; Gibbs & Elliott, 2015; Kumas et al., 2021; Sharma et al., 2015). These needs should inform the design of technological tools and research-based teaching interventions, for example, the potential usage of artificial grammar learning in teaching students with DD (Folia et al., 2008, 2010, 2023), or the integration of timing skills and duration perception as learning aids for students with DD (Catronas et al., 2023; Liapi et al., 2024; Torres et al., 2025).

On a theoretical level, research should develop nuanced frameworks that connect disciplinary knowledge, teacher cognition, and inclusive pedagogy across diverse educational and cultural contexts—moving from abstract models to practical, context-sensitive approaches.

## 5. Conclusions

The present systematic review highlights the critical role of EFL teachers’ perceptions and preparedness in supporting students with DD. Our findings reveal mixed perceptions among EFL teachers toward DD. In particular, while many EFL teachers are aware of DD and express a willingness to support learners with DD, they often lack the necessary training and resources to do so effectively. Similarly, with respect to attitudes and perspectives, while many EFL teachers acknowledge the importance of addressing DD, prevailing misconceptions and limited understanding often result in lower expectations and inadequate support for affected students. Lastly, related to teachers’ preparedness, significant gaps in formal training and a lack of practical strategies focused on inclusive EFL instruction were found. To bridge the gap between knowledge and practice, there is an urgent need for future research to focus on developing and evaluating targeted professional development programs and evidence-based teaching interventions. Future policies should strive to strengthen teacher training and provide practical resources. These are essential steps toward a more inclusive and effective EFL learning environment for students with DD.

## Figures and Tables

**Table 1:** Descriptive information related to the reviewed articles.

Year	Authors	Country	Participants	Research Tools	Main Findings
2011	Lemperou, L., Chostelidou, D., & Griva, E.	Greece	*n* = 94	Self-constructed questionnaires	Teachers are aware of DD but lack the knowledge to create suitable teaching approaches.
2017	Cimermanová, I.	Slovakia	*n* = 243 (187 in-service and 56 pre-service EFL teachers)	Self-constructed questionnaires	Teachers lack any special training.
2018	Nijakowska, J., Tsagari, D., & Spanoudis, G.	Greece, Cyprus, and Poland	*n* = 546	DysTEFL-NAQ-R	Training may enhance teachers’ skills and knowledge for supporting DD learners.
2019	Fekih, M.	Algeria	*n* = 42 *	Self-constructed questionnaires, interviews, and focus group	The majority of teachers is largely unfamiliar with DD and need training.
2019	Indrarathne, B.	Sri Lanka	*n* = 129 *	Self-constructed questionnaires and interviews	Teachers lack training and inclusive language teaching practices.
2019	Babuder, M. K. & Jazbec, S.	Slovenia	*n* = 96 (pre- and in-service FL teachers of German or English)	Self-constructed questionnaires	Teachers’ limited experience of teaching FL to DD students.
2020	Haggag, H. M., & Bakr, E. M. E.	Egypt	*n* = 99 *	Self-constructed questionnaires and online reflection journal of the participants via Blogger	Teachers need enhanced training in intervention techniques and assessment practices.
2020	Kalsoom, T., Mujahid, A. H., & Zulfqar, A.	Pakistan	*n* = 328 *	Self-constructed questionnaires	Teachers need enhanced support and training on strategies.
2020	Tobbi, S.	Algeria	*n* = 81 *	Self-constructed questionnaires	Teachers’ poor awareness of DD and training.
2021	Kumas, Ö. A., Dodur, H. M. S., & Yazicioglu, T.	Turkey	*n* = 400 *	TBALQ and interviews	Teachers lack awareness about DD and training. Teaching approaches.
2022	Mohammad, Z. A.	Iraq	*n* = 34 *	Two self-constructed questionnaires	Teachers lack understanding of DD and training.
2022	Nijakowska, J.	Greece, Slovenia, and Poland	*n* = 69 *	FLIPD	Intensive training may enhance teachers’ preparedness for inclusive practices.
2023	Nushi M., & Eshraghi, M.	Iran	*n* = 84 *	KBDDS	EFL teachers’ awareness of DD is low, but teachers are willing to learn more.
2023	Vizhi, P. K., & Rathnasabapathy, M.	India (Chennai District)	*n* = 19 *	Self-constructed questionnaires	Difficulties DD students face, and methods teachers use.
2024	Oskwarek, A., Polok, K., & Przybysz-Zaremba, M.	Poland (Upper Silesia Province)	*n* = 75 *	Self-constructed questionnaires	Teaching procedures and assessment methods used for DD students. Teachers’ have inadequate training and resources.
2024	Özçelik, A. E., & Elverici, S. E.	Turkey	*n* = 180 *	DysTEFL-NAQ-R	Teachers’ lack of awareness and knowledge about DD.

Note: DysTEFL-NAQ-R, DysTEFL-Needs Analysis Questionnaire Revised; TBALQ, The Development of the Teachers’ Beliefs About Literacy Questionnaire; FLIPD, Perceptions about Inclusive Practices in Teaching Foreign Languages to Dyslexic Language Learners; KBDDS: The Knowledge and Beliefs about Developmental Dyslexia Scale. * The number of participants in these studies consists exclusively of EFL teachers.

**Table 2:** Foreign language teachers’ awareness regarding developmental dyslexia.

Year	Authors	Country	Findings on EFL Teachers’ Awareness
2011	Lemperou, L., Chostelidou, D., & Griva, E.	Greece	51% of EFL teachers were aware of DD and its challenges; 45% had limited knowledge; 4% were moderately familiar.
2018	Nijakowska, J., Tsagari, D., & Spanoudis, G.	Greece, Cyprus, and Poland	94.4% of teachers felt they needed more information on teaching DD learners; 62.5% were determined to learn more.
2019	Fekih, M.	Algeria	Most Algerian EFL teachers had a poor understanding of DD.
2019	Indrarathne, B.	Sri Lanka	94% of teachers did not have a clear understanding of DD.
2019	Babuder, M. K. & Jazbec, S.	Slovenia	Teachers had theoretical awareness of DD, but few had direct experience teaching DD students.
2020	Haggag, H. M., & Bakr, E. M. E.	Egypt	Participants were aware only of the basic concepts of SLD.
2020	Kalsoom, T., Mujahid, A. H., & Zulfqar, A.	Pakistan	Most participants lacked awareness of students’ SLD.
2020	Tobbi, S.	Algeria	Only 11.11% of teachers were aware of DD. Overall awareness was poor.
2022	Mohammad, Z. A.	Iraq	97% of respondents knew something about DD.
2022	Nijakowska, J.	Greece, Slovenia, and Poland	63.6% rated their knowledge of DD as average; 31.9% rated it as poor; 11.6% rated it as good.
2023	Nushi M., & Eshraghi, M.	Iran	The majority of teachers lacked adequate knowledge of DD.
2024	Oskwarek, A., Polok, K., & Przybysz-Zaremba, M.	Poland	Most FL teachers were aware of DD.
2024	Özçelik, A. E., & Elverici, S. E.	Turkey	8.9% of teachers did not believe English teachers should have high expectations for DD learners, suggesting a lack of awareness.

**Table 3:** EFL teachers’ training on developmental dyslexia.

Year	Authors	Country	Findings on Teacher Training
2011	Lemperou, L., Chostelidou, D., & Griva, E.	Greece	87% expressed strong interest in INSET training; only 2.1% received in-school training.
2017	Cimermanová, I.	Slovakia	34.9% had no special training.
2018	Nijakowska, J., Tsagari, D., & Spanoudis, G.	Greece, Cyprus, and Poland	90.7% showed some interest in training.
2019	Indrarathne, B.	Sri Lanka	None of the teachers had received training.
2019	Babuder, M. K. & Jazbec, S.	Slovenia	63.6% of in-service teachers and 40.4% of pre-service teachers found two days of training appropriate.
2020	Tobbi, S.	Algeria	93.83% of teachers believed they would benefit from training programs.
2021	Kumas, Ö. A., Dodur, H. M. S., & Yazicioglu, T.	Turkey	95% of teachers had not received training.
2022	Mohammad, Z. A.	Iraq	52.9% of respondents needed training, but only 8.8% had participated in related courses.
2022	Nijakowska, J.	Greece, Slovenia, and Poland	39.1% of teachers had received some previous training, implying 60.9% had not.
2023	Nushi M., & Eshraghi, M.	Iran	The majority of teachers wished to receive training on DD.
2024	Özçelik, A. E., & Elverici, S. E.	Turkey	41.1% showed interest in additional training; 37.2% were committed to enrolling in professional development courses.

## Data Availability

No new data were created.
